# Implementation of the Maynard-Based Risk Assessment Model for Venous Thromboembolism Inpatient Prophylaxis: A Before-and-After Study

**DOI:** 10.3390/healthcare13243204

**Published:** 2025-12-08

**Authors:** Belisa Marin Alves, Raquel Pereira Vieira, Larissa Luma Tomasi Febras, Mauricio Santiago Soper, Jonas Michel Wolf, Vania Rohsig, Sidiclei Machado Carvalho, Cássia Cristine Damasio de Lima, Cintia Lazzari, Daniel Luft Machado, Luiz Antônio Nasi, Marcelo Basso Gazzana

**Affiliations:** 1 Graduate Program in Pulmonary Sciences, Federal University of Rio Grande do Sul (UFRGS), Porto Alegre 91501-970, RS, Brazil; belisa.alves@hmv.org.br (B.M.A.); mbgazzana@hcpa.edu.br (M.B.G.); 2 Hospital Moinhos de Vento (HMV), Porto Alegre 90035-000, RS, Braziljonas.wolf@hmv.org.br (J.M.W.); vania.rohsig@hmv.org.br (V.R.); sidiclei.carvalho@hmv.org.br (S.M.C.); cassia.lima@hmv.org.br (C.C.D.d.L.); cintia.lazzari@hmv.org.br (C.L.); daniel.machado@hmv.org.br (D.L.M.);

**Keywords:** risk assessment, multifaceted interventions, electronic tool, prophylaxis, venous thromboembolism

## Abstract

**Highlights:**

**What are the main findings?**

- The implementation of the Maynard risk assessment model impaired the application of prophylactic measures in orthopedic patients.
- The Maynard risk assessment model showed limited discriminative performance.

**What is the implication of the main finding?**

- There is a need to enhance the discriminatory performance of the Maynard risk assessment model.

**Abstract:**

**Background/Objectives:** Venous thromboembolism (VTE) is a common and potentially fatal condition in hospitalized patients. Although appropriate risk assessment and prophylaxis reduce VTE events, preventive measures remain underutilized. This study aimed to evaluate the effectiveness of an electronic risk stratification tool within multifaceted interventions for implementing VTE prophylaxis protocols in adult clinical and surgical patients at Hospital Moinhos de Vento, Brazil. **Methods:** A prospective before-and-after hospital-based study was conducted from 2017 to 2019, including 772 patients admitted to clinical and surgical units for over 48 h. The electronic tool based on the Maynard risk assessment model classified patients’ VTE risk. Padua and Caprini scores served as reference standards for clinical and surgical patients, respectively. Prophylaxis was considered adequate if it complied with institutional protocols. **Results:** Globally, the Maynard model classified 0.9% as low risk, 76.4% intermediate, and 22.7% high risk, differing notably in orthopedic surgical patients. Overall prophylaxis adequacy was 69.3%, with no significant difference between phases. Orthopedic surgical patients showed a significant decrease in prophylaxis adequacy in phase 2 (*p* = 0.02). **Conclusions:** The Maynard model underestimated high-risk classification compared to Padua and Caprini scores, especially in orthopedic surgical patients. Implementation of the electronic tool alongside multifaceted interventions did not improve prophylaxis adequacy.

## 1. Introduction

Venous thromboembolism (VTE) is a condition that encompasses both deep vein thrombosis (DVT) and pulmonary embolism (PE), representing different manifestations of the same disease spectrum. DVT results from the formation of a blood clot, usually originating in the deep veins of the lower limbs (iliac, femoral, and popliteal veins), which can cause a potentially severe and fatal complication when the clot detaches and is carried through the bloodstream, becoming lodged and obstructing the pulmonary arterial circulation, known as PE, which may lead to acute right ventricular dysfunction, obstructive shock, and death [1].

VTE is globally recognized as the third most common cause of acute cardiovascular syndrome and one of the preventable etiologies of in-hospital death [2,3]. In Brazil, according to data from the Ministry of Health collected between 2010 and 2021, the number of hospitalizations related to VTE exceeded 520,000, with more than 67,000 deaths between 2010 and 2019, constituting a high mortality rate [4].

There are numerous risk factors associated with VTE, such as surgeries, active cancer, decompensated heart failure, acute stroke, acute infections, previous VTE episodes, pregnancy-puerperal cycle, and presence of central venous catheter, many of which are present in hospitalized patients [1,2]. VTE events are considered highly preventable; therefore, regulatory agencies and medical societies recommend individualized risk assessments and prophylaxis for each hospitalized patient, practices reflected in hospital quality indicators [5,6,7]. Evidence clearly shows that adequate pharmacological prophylaxis significantly reduces the incidence of VTE and mortality in hospitalized patients [8]. Despite strong recommendations, VTE prevention remains underutilized in clinical practice [9,10]. Various strategies have been employed to improve adherence to VTE prophylaxis. The use of multifaceted interventions (consultative materials, multidisciplinary educational meetings, patient-directed actions, audits, feedback, electronic risk stratification scales, electronic reminders, etc.) has demonstrated efficacy in randomized clinical trials, although results are heterogeneous and dependent on intervention type [11].

Thus, risk stratification scores for VTE, such as those proposed by Caprini [12] and Padua [13], have been developed and validated for use in surgical and clinical patients, respectively [14]. However, the scores results depend on the sum of multiple factors, which requires time and reduces adherence. The risk stratification model proposed by Maynard [15,16] does not require calculation and can be used to evaluate both clinical and surgical patients through a single integrated tool.

Therefore, the objective of this study was to compare the effectiveness of the Maynard model with the Padua and Caprini scores for VTE risk stratification in hospitalized clinical and surgical patients, as well as to assess its impact on the adequacy of prophylaxis for this condition.

## 2. Materials and Methods

This prospective, non-randomized before-and-after study was conducted at a private hospital in southern Brazil, located in Porto Alegre, Rio Grande do Sul, with 485 beds. The study aimed to evaluate the effectiveness of an electronic tool for VTE risk stratification in hospitalized patients, between August 2017 and December 2019.

The research was conducted in two phases: Phase 1 took place 12 months prior to the implementation of the electronic decision-support tool for physicians, designed to classify patients’ VTE risk during hospitalization based on the Maynard risk stratification model. The model allows empirical stratification of patients’ risk and recommends appropriate prophylactic measures. It was mandatory to complete upon the patient’s initial medical prescription and remained available for review throughout the hospital stay as needed. Phase 2 occurred 12 months after the tool’s implementation. The electronic tool remained active during the 12-month interval between the two phases.

Adults aged 18 years or older admitted to clinical or surgical units with a minimum hospital stay of 48 h were included. Exclusion criteria were pregnancy, puerperium, admission to adult intensive care units, use of therapeutic anticoagulants prior to admission or started at admission, suspicion or diagnosis of pulmonary embolism (PE) or deep vein thrombosis (DVT) at admission, hospital stays exceeding 90 days, incomplete medical records, and inability to respond to interviews due to cognitive impairment. Patients were categorized into three groups in each phase: Group 1—Clinical patients; Group 2—Orthopedic surgical patients; and Group 3—Non-orthopedic surgical patients.

VTE risk scores (Padua for clinical patients and Caprini for surgical patients) were also calculated based on patient data. Both models are based on a cumulative point-system. They aggregate individual risk factors, such as age, comorbidities, immobilization, prior history of venous thromboembolism, surgery, cancer, and obesity, among others, to generate a numerical score that reflects the patient’s estimated probability of VTE and guides prophylaxis strategy, representing the current standard of care.

Face-to-face interviews were conducted using a structured questionnaire focusing on risk factors for DVT and PE among clinical, orthopedic, and non-orthopedic surgical patients. Demographic data (sex, age, weight, body mass index [BMI]), comorbidities (stroke, renal failure, myocardial infarction, cancer, thrombophilia, prior DVT/PE), contraindications to therapy (ulcers, prior bleeding episodes), and laboratory parameters (international normalized ratio [INR], platelet count) were collected. Additional information on hospital stay duration, initiation, type, dose, and frequency of prophylaxis prescription, use of anticoagulants and antiplatelet agents, and medical specialty were obtained through active medical record review.

The algorithm was developed according to the institutional clinical protocol for VTE, which used the Maynard risk stratification as the basis for risk assessment and prophylaxis prescription recommendations according to the selected risk level [15,16]. This tool was made available to the hospital clinical staff through a module integrated into the electronic medical record system. It enables the prescribing physician to stratify VTE risk at the time of the initial prescription. The physician classifies the patient into one of three risk categories (low, moderate, or high), without the need to individually select isolated risk factors, or calculate a score. Completion of the tool is mandatory and it remains available for review throughout the hospitalization if needed. Additionally, contraindications to pharmacological prophylaxis must be documented to justify non-prescription. The primary outcome measure was the “VTE Prophylaxis Appropriateness Rate,” as defined by the Joint Commission International Library [7].

Sample size calculation was performed using WinPepi software version 11.65, considering an expected absolute increase of 15% in prophylaxis adequacy, 80% power, and 5% two-sided significance level, plus 5% to account for possible losses. Based on institutional baseline adequacy estimates and data from Leal et al. [17], 170 patients per phase were required for clinical patients, and 105 patients per phase for each surgical subgroup, totaling 380 eligible patients.

Data entry was conducted in Microsoft Excel, and double entry validation and data review were performed using SPSS version 25.0 (SPSS Inc., Chicago, IL, USA). Categorical variables were presented as absolute and relative frequencies. Quantitative variables were expressed as means ± standard deviation or medians and interquartile ranges, depending on normality (Shapiro–Wilk test). The Kruskal–Wallis test with Dunn’s post hoc was used to compare medians, while categorical variables were compared using Pearson’s chi-square test. A significance level of 5% was adopted for all analyses.

The study protocol was approved by the Ethics Committee of the Hospital Moinhos de Vento Education and Research Institute (CAAE: 30191514.7.0000.5330). The study adhered to the Brazilian National Health Council Resolution 466/12 guidelines. Informed consent was obtained from all participants, and confidentiality was maintained throughout the study.

## 3. Results

This study included a total of 943 patients interviewed, with 497 in phase 1 and 446 in phase 2 (pre- and post-intervention, respectively). A total of 171 patients were excluded: 107 in phase 1 and 64 in phase 2. The reasons for exclusion were as follows: 91 due to hospitalization shorter than 48 h, 11 due to hospitalization longer than 90 days, 51 on anticoagulant therapy, and 18 duplicate samples.

**Figure 1:**
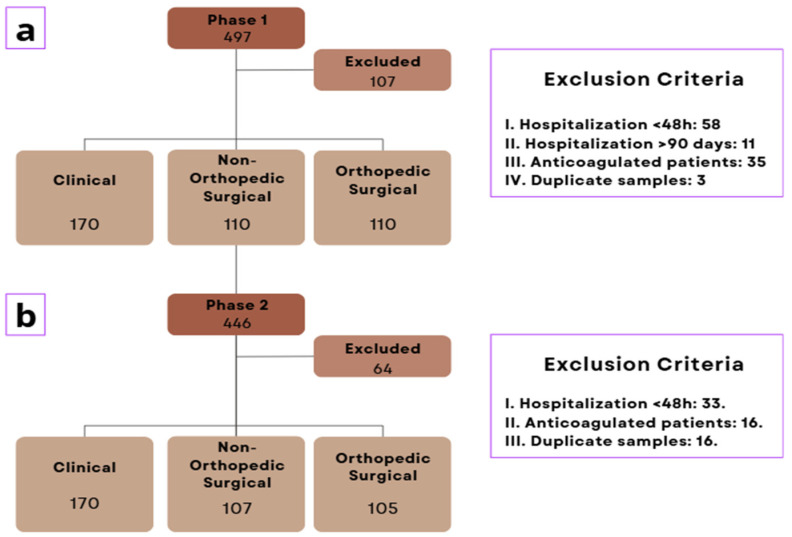
Inclusion flowchart where (**a**): Phase 1 sample; (**b**) Phase 2 sample.

**Figure 2:**
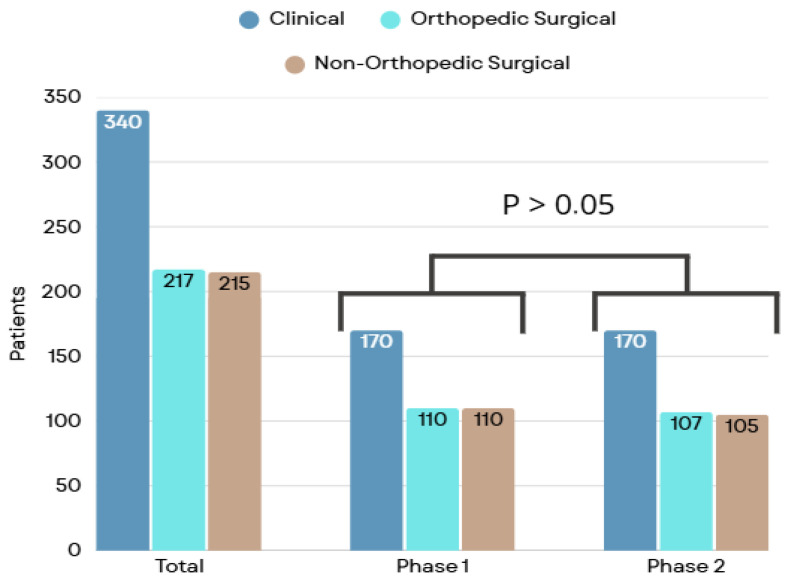
Patient classification by specialty group.

Of the total, 772 participants were included, with 390 (50.5%) in phase 1 and 382 (49.5%) in phase 2. The inclusion flowchart and the proportions of clinical, orthopedic surgical, and non-orthopedic surgical groups are presented in Figure 1 and Figure 2, respectively.

Globally, the sample consisted of patients with a median age of 63 years, with a predominance of females. The median [IQR] age was higher in the clinical group (phase 1: 64.0 [46.0–79.0], phase 2: 67.0 [51.0–78.0] years) compared to the non-orthopedic surgical group (phase 1: 56.0 [43.0–66.0], phase 2: 59.0 [41.0–70.0] years) (*p* < 0.01 and *p* < 0.01, respectively) (Table 1). Female sex was predominant, accounting for 59.8% of cases in phase 1 and 51% in phase 2, as was white ethnicity, representing more than 95% of the sample in both phases. Comparisons between groups were performed using the chi-square (χ^2^) test, and the resulting *p*-value greater than 0.05 was considered indicative of no statistically significant difference between groups.

When analyzing individual risk factors, the most frequent overall were smoking (current or former), active cancer, and the presence of a central venous catheter (Table 2). Notably, in phase 1, recent cancer was significantly associated with the non-orthopedic surgical group, reaching 34.5%. This trend persisted in phase 2, increasing to 47.6%, reinforcing the importance of this condition as a key factor in the non-orthopedic surgical context. Conversely, the use of a central venous catheter showed a consistent association with the clinical group, with rates of 27.2% and 30.6% in phases 1 and 2, respectively.

**Figure 3:**
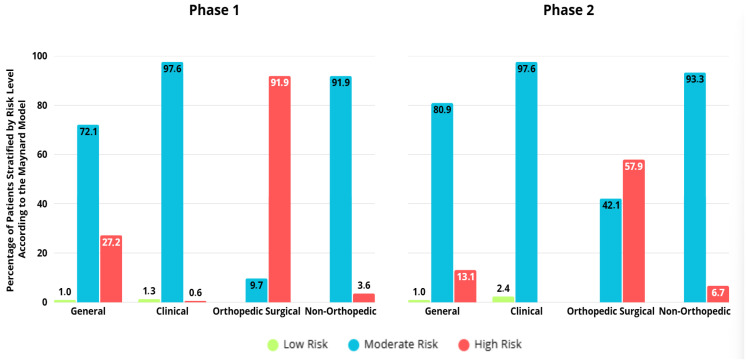
Stratification of venous thromboembolism risk according to Maynard, by group and study phase.

Globally, the Maynard risk stratification classified patients as having intermediate risk in more than 70% of cases, and low risk in less than 5% of cases, across both phases and in most patient groups. In this sense, the stratification appears to be poorly discriminative, and could lead to less accurate risk assessment in clinical settings, mainly due to the fact that it does not allow physicians to properly distinguish between patients with different risk factors (Figure 3).

In phase 1, 90 clinical participants (52.9%) where classified as high VTE risk according to the Padua score, in contrast to only 1 participant (0.6%) by the Maynard model, once again demonstrating the poor performance of the Maynard model, which could consequently lead to the under prescription of prophylactic measures. On the other hand, high-risk classification by the Maynard model was predominantly associated with the orthopedic surgical group (91.8%), while intermediate-risk categorization was more prevalent in the clinical (97.6%) and non-orthopedic surgical (96.4%) groups (*p* < 0.01). High-risk classification by the Caprini score was assigned to 207 participants (94.1%), with no significant differences between groups during phase 1 (Table 3).

In phase 2, 125 participants (73.5%) were classified as high risk by the Padua score, while 69 participants (18.1%) received the same classification by the Maynard model. Furthermore, high-risk classification by the Maynard model was predominantly associated with the orthopedic surgical group (57.9%), whereas intermediate-risk categorization was prevalent in the clinical (97.6%) and non-orthopedic surgical (93.3%) groups (*p* < 0.01). In phase 2, high-risk classification by the Caprini score was assigned to 192 participants (90.6%), showing a significant association with the orthopedic surgical group (96.3%), compared to low and moderate risk classifications associated with the non-orthopedic surgical group (3.8% and 11.4%, respectively) (*p* = 0.01) (Table 3).

A significant difference was observed in the classification of orthopedic patients by the Maynard stratification between phases, with fewer patients classified as high risk in phase 2 compared to phase 1 (*p* < 0.01). As low-risk patients were infrequent in both phases, a greater number of patients were consequently classified as intermediate risk in phase 2 compared to phase 1 (*p* < 0.01).

**Figure 4:**
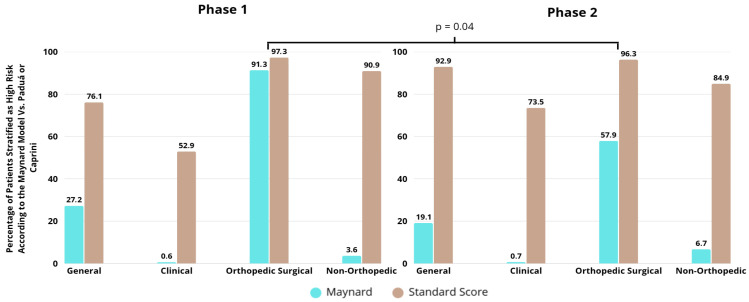
Comparison between Maynard stratification and comparator in relation to the classification of patients as high risk of venous thromboembolism by group and study phase.

Maynard stratification was compared with the standard score, the Padua score for medical patients and the Caprini score for surgical patients. In most contexts, Maynard stratification underestimates risk, not indicating a high risk of more than 40% when analyzed with a standard comparator. For orthopedic patients, Maynard stratification performs better than in the clinical and non-orthopedic surgical groups, although in phase 2, its effectiveness was lower even in orthopedic surgical patients (*p* = 0.04) (Figure 4).

**Figure 5:**
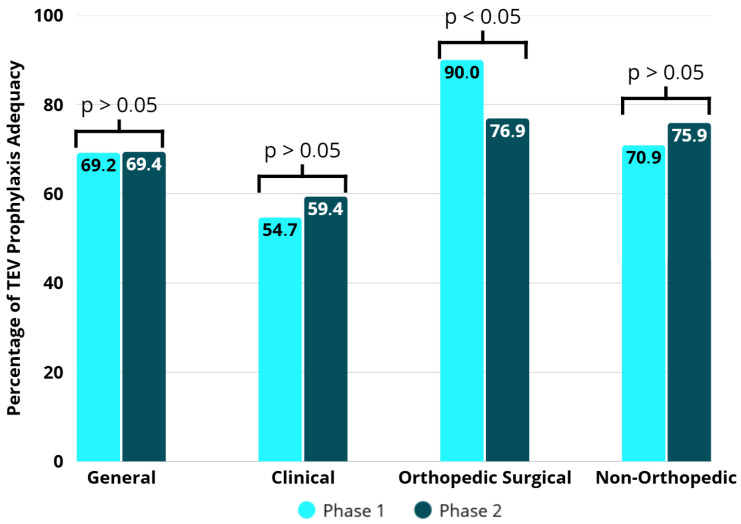
Adequacy of VTE prophylaxis in the groups by study phases.

According to the assessment of VTE prevention in relation to what is recommended in the institutional protocol, 535 (69.0%) participants showed prophylactic adequacy in both phases, 270 (69.2%) during phase 1 and 265 (69.4%) during phase 2, with no significant difference (*p* = 0.99) (Table 4). In phase 1, prophylactic adequacy was more associated with orthopedic surgical participants (90.0%), while in phase 2, prophylactic adequacy was higher in orthopedic surgical (76.9%) and non-orthopedic (77.9%) groups. There was no difference between phase 1 and phase 2 in the clinical and non-orthopedic surgical groups (*p* = 0.44 and *p* = 0.31, respectively). It should be clarified that the tool’s stable performance was likely attributable to its low sensitivity. However, in the orthopedic group, there was a significant reduction in the adequacy of prophylaxis after the intervention (*p* = 0.02) (Figure 5).

Regarding the forms of prophylaxis, almost all patients used pharmacological prophylaxis (enoxaparin, unfractionated heparin, fondaparin, dabigatran, rivaroxaban, or apixaban). Only 2 patients reported using non-pharmacological prophylaxis (venous return pump).

## 4. Discussion

This study evaluated the use of an electronic tool for risk estimation based on the Maynard risk stratification model and its effect on the appropriateness of VTE prophylaxis. The research involved over 700 hospitalized patients in clinical and surgical wards (excluding patients admitted to the intensive care unit), who were pre-stratified in a balanced manner into three groups: clinical, orthopedic surgical, and non-orthopedic surgical. The implementation of the tool did not demonstrate good performance for risk stratification when compared to guideline-recommended scores and did not improve the rates of appropriate VTE prophylaxis prescription.

Hospitalized patients are particularly prone to VTE events, and this population was the focus of the present study [17]. The sample consisted of patients with a median age of around 60 years, a slight predominance of females, and the presence of classical risk factors for VTE, including active cancer and central venous catheter use. Previous studies conducted in similar settings have shown comparable patient profiles [17,18,19].

Risk stratification is a fundamental step in VTE prevention strategies [20]. Although physicians may estimate risk empirically, clinical guidelines recommend the use of validated VTE risk assessment models, the most used being the Padua and Caprini scores, both derived and validated in independent cohorts [6,7]. Other scores and models exist (Geneva, IMPROVE, Kucher, etc.), but a recent meta-analysis revealed significant heterogeneity among strategies, with sensitivities ranging from 12% to 100% and specificities from 7.2% to 100% [14].

In our study, more than 75% of patients were categorized as high risk for VTE, reflecting both the complexity of the institution and the exclusion of patients hospitalized for less than 48 h (who are generally considered low risk for VTE). Risk assessed using the standard comparator scores (Padua and Caprini) remained stable across both phases and all groups, demonstrating that the patient risk profiles were comparable throughout the study.

The use of scoring systems in daily practice remains a challenge due to the multitude of options, the complexity of the models, and the time required for completion, all of them factors that contribute to low adherence. A meta-analysis highlighted significant variability in the implementation of the Caprini score [21]. A recent survey of American physicians revealed that only 35% used risk scores to guide prophylaxis prescriptions [9]. In this context, simplified models have been proposed, including the Maynard risk stratification model, which is less labor-intensive. This model is descriptive, meaning that risk factors are listed under each category without the need for a calculated score or a minimum number of items to define a risk level [16].

In the present study, the Maynard stratification model classified an excessive number of patients as intermediate risk: 72.1% in phase 1 and 80.9% in phase 2. This limits the model’s ability to distinguish VTE risk levels within the patient population. However, our findings were similar to those in the original Maynard study, which reported 4% low risk, 84% intermediate risk, and 12% high risk [16].

Prophylactic methods should be applied according to the patient’s risk level [22]. Several studies have consistently demonstrated the benefit of pharmacologic thromboprophylaxis in higher risk levels, while low-risk patients do not require pharmacologic or mechanical prophylaxis, but early ambulation is recommended [23]. On the other hand, different pharmacologic regimens should be chosen based on patient profiles (including comorbidities), VTE risk, and contraindications to anticoagulants. Both under-prescription (when indicated) and over-prescription (when not indicated) are considered inappropriate [8]. In our study, prophylaxis was inappropriate in approximately 30% of patients, mainly due to underuse when indicated. In clinical practice, the adequacy of prophylaxis varies from 50% to 95% [5]. In Maynard’s original study, the adequacy rate improved from 58% to 93% over three years following implementation of the model [15]. This suggests that maintaining the risk model in clinical practice has the potential to improve the adequacy of prophylaxis. However, co-interventions were not controlled for, making it impossible to attribute improvements solely to the Maynard model. In our study, the Maynard model did not demonstrate effectiveness in improving VTE prophylaxis prescription rates according to the institutional protocol.

It is important to highlight that the Maynard stratification table is informative only; the electronic tool implemented did not require users to confirm whether each risk factor was present. This allows for a degree of subjectivity in risk estimation, as physicians select the risk category based on their impression, which may limit the effectiveness of the strategy compared to usual clinical practice, in this sense, our findings seem to support this notion.

The use of appropriate prophylaxis follows different patterns among patient groups [24]. Typically, orthopedic patients present higher rates of correct prescription compared to medical and non-orthopedic surgical patients, mainly due to the high thromboembolic risk associated with fracture and/or hip and knee prosthesis surgeries. In this context, there is a higher number of thromboembolic events (which leads physicians to be more proactive regarding prophylaxis), stronger evidence of benefit, better-defined protocols, and greater uniformity of clinical practices [25]. In the present study, orthopedic surgical patients received adequate prophylaxis in 90% and 76.9% of cases in phases 1 and 2, respectively. Notably, there was a significant decrease in prophylaxis adequacy after the implementation of the electronic tool. The risk profile according to the standard Caprini score (which is the standard scoring system for orthopedic patients) did not differ between phases (96.3% in phase 1 and 97.3% in phase 2). However, according to the Maynard risk stratification, there was a significant reduction in the proportion of patients classified as high risk (91.8% in phase 1 and 57.9% in phase 2). It can be inferred that inadequate stratification in relation to the standard comparator score led physicians not to prescribe thromboprophylaxis to patients who had an indication for it, resulting in inappropriate management.

Although various strategies have been proposed to improve the implementation of VTE prophylaxis protocols in daily clinical practice, one of the greatest challenges in healthcare persists: translating research evidence into clinical practice, which often requires cultural and behavioral changes among professionals [26,27]. A commonly held view is that multifaceted interventions are more effective than single-component interventions [27]. A recent meta-analysis in the context of VTE prevention demonstrated the benefit of multiple interventions, particularly electronic alerts [28]. In our study, the addition of an electronic risk stratification tool to other educational interventions did not improve prescription adequacy when comparing the periods before and after implementation, showing a significative decrease in adequacy of orthopedic patients, mainly due to the poor discriminative performance of the Maynard risk stratification. As a future perspective, artificial intelligence-based tools are now being tested in this context and have shown promising results [29].

This study has some limitations. First, the sample was non-randomized and based on convenience, using a before-and-after design, which may have led to imbalances in patient characteristics between phases. However, the total number of patients was robust, and the sample size was stratified by group (clinical, orthopedic surgical, and non-orthopedic surgical), avoiding group size imbalances. Second, there were few records of non-pharmacologic prophylaxis, possibly due to issues with availability, lack of awareness, medical records or logistical difficulties, which may have affected adherence to the institutional protocol. Third, the two-year interval between study phases may have led to changes in clinical practice, although institutional recommendations remained the same. It’s worth noting that the hospital operates with an open medical staff (physicians with diverse backgrounds who work at other institutions with potentially different VTE prophylaxis guidelines) and runs a residency program with yearly turnover. These factors may have influenced physician behavior regarding prophylaxis between phases.

Finally, risk categories were compared to reference scores (as intermediate outcomes), but actual clinical events such as DVT and PE were not evaluated which would be the most accurate way to validate the effectiveness of risk stratification.

## 5. Conclusions

The implementation of an electronic tool for risk stratification based on the Maynard model underestimated VTE risk in a large proportion of patients when compared to standard scoring systems. Additionally, the use of this technology did not improve the adequacy of VTE prophylaxis; in fact, prophylactic measures worsened among orthopedic patients—likely due to the misclassification of risk. Further studies are needed to validate these findings in different populations and institutions, as well as to refine the Maynard stratification model by enhancing its discriminatory power between risk groups, particularly within the intermediate-risk category.

## Figures and Tables

**Table 1:** Demographic characteristics by group and study phase.

Phases	Variables	Total (*n* = 772)	Clinical (*n* = 340)	Orthopedic Surgical (*n* = 217)	Non-Orthopedic Surgical (*n* = 215)	*p*-Value ^1^
Phase 1	*n*	390	170	110	110	
	Age (years)	62.0 [46.0–76.0]	64.0 [46.0–79.0]	63.5 [47.0–76.0]	56.0 [43.0–66.0]	<0.01
	Female Sex	237 (59.8)	107 (62.9)	70 (63.6)	60 (54.5)	0.28
	White Color Ethnicity	379 (97.1)	167 (98.2)	108 (98.2)	104 (94.5)	0.75
	BMI (kg/m^2^)	26.1 [23.4–29.0]	25.1 [22.6–28.6]	26.5 [24.3–29.3]	26.4 [23.8–30.4]	<0.01
Phase 2	*n*	382	170	107	105	
	Age (years)	64.0 [45.0–75.0]	67.0 [51.0–78.0]	61.0 [44.5–75.0]	59.0 [41.0–70.0]	<0.01
	Female Sex	195 (51.0)	90 (52.9)	58 (54.2)	47 (44.8)	0.31
	White Color Ethnicity	382 (100.0)	170 (100.0)	107 (100.0)	105 (100.0)	0.99
	BMI (kg/m^2^) ^2^	26.4 [23.1–30.7]	25.7 [22.2–30.0]	26.8 [24.3–30.7]	26.7 [23.8–31.4]	0.09

^1^ *p*-value corresponding to comparisons between groups within the same phase. ^2^ Body Mass Index.

**Table 2:** Venous thromboembolism risk factors by group and phase of the study.

Phases	Variables	Total (*n* = 772)	Clinical (*n* = 340)	Orthopedic Surgical (*n* = 217)	Non-Orthopedic Surgical (*n* = 215)	*p*-Value ^1^
Phase 1	*n*	390	179	110	110	
	Previous PE/DVT ^2^	19 (49.0)	11 (6.5)	6 (5.5)	2 (1.8)	0.19
	Active cancer	80 (20.5)	36 (21.2)	6 (5.5)	38 (34.5)	<0.01
	Hormone use	40 (10.3)	18 (10.6)	14 (12.7)	8 (7.3)	0.41
	CVC ^3^	76 (19.5)	46 (27.2)	8 (7.3)	22 (20.0)	<0.01
	Smoking (current/or former)	119 (32.0)	51 (30.0)	26 (23.7)	44 (40.0)	0.08
	Fracture/Trauma	23 (5.9)	6 (3.5)	17 (15.5)	0 (0.0)	<0.01
	Immobility	233 (59.7)	103 (60.6)	73 (66.4)	57 (51.8)	0.08
Phase 2	*n*	382	170	107	105	
	Previous PE/DVT ^2^	39 (10.2)	21 (12.4)	9 (8.4)	9 (8.6)	0.46
	Active cancer	110 (28.8)	48 (28.2)	12 (11.2)	50 (47.6)	<0.01
	Hormone use	36 (9.4)	14 (8.2)	11 (10.3)	11 (10.5)	0.77
	CVC ^3^	87 (22.8)	52 (30.6)	12 (11.3)	23 (21.9)	<0.01
	Smoking (current or former)	146 (38.2)	72 (42.4)	30 (28.0)	44 (41.9)	0.04
	Fracture/Trauma	29 (7.6)	8 (4.7)	19 (17.8)	2 (1.9)	<0.01
	Immobility	219 (57.3)	92 (54.1)	76 (71.0)	51 (48.6)	<0.01

^1^ *p*-value corresponding to comparisons between groups within the same phase. ^2^ Pulmonary embolism/Deep vein thrombosis. ^3^ Central venous catheter.

**Table 3:** VTE risk stratification according to the Padua, Maynard, and Caprini models by group and study phase. ^1^ *p*-value corresponding to comparisons between groups within the same phase.

Phases	Variables	Total (*n* = 772)	Clinical (*n* = 340)	Orthopedic Surgical (*n* = 217)	Non-Orthopedic Surgical (*n* = 215)	*p*-Value ^1^
Phase 1 (*n* = 390)	Pádua					
	Low Risk	80 (47.1)	80 (47.1)	-	-	-
	High Risk	90 (52.9)	90 (52.9)	-	-	
	Maynard					
	Low Risk	3 (0.8)	3 (1.8)	0	0	<0.01
	Intermediate Risk	281 (72.1)	166 (97.6)	9 (8.2)	106 (96.4)	
	High Risk	106 (27.2)	1 (0.6)	101 (91.8)	4 (3.6)	
	Caprini					
	Very Low Risk	0	-	0	0	0.08
	Low Risk	0	-	0	0	
	Intermediate Risk	13 (5.9)	-	3 (2.7)	10 (9.1)	
	High Risk	207 (94.1)	-	107 (97.3)	100 (90.9)	
Phase 2 (*n* = 382)	Pádua					
	Low Risk	45 (26.5)	45 (26.5)	-	-	-
	High Risk	125 (73.5)	125 (73.5)	-	-	
	Maynard					
	Low Risk	4 (1.0)	4 (2.4)	0	0	<0.01
	Intermediate Risk	309 (80.9)	166 (97.6)	45 (42.1)	98 (93.3)	
	High Risk	69 (18.1)	0	62 (57.9)	7 (6.7)	
	Caprini					
	Very Low Risk	0	-	0	0	0.01
	Low Risk	4 (1.9)	-	0	4 (3.8)	
	Intermediate Risk	16 (7.5)	-	4 (3.7)	12 (11.4)	
	High Risk	192 (90.6)	-	103 (96.3)	89 (84.8)	

**Table 4:** Adequacy of VTE prophylaxis used in the groups by phase.

Group	Phase 1 (*n* = 390)	Phase 2 (*n* = 382)	*p*-Value
General			
Not Adequate	120 (30.8)	117 (30.6)	0.99
Adequate	270 (69.2)	265 (69.4)	
Clinical			
Not Adequate	77 (45.3)	69 (40.6)	0.44
Adequate	93 (54.7)	101 (59.4)	
Orthopedic Surgical			
Not Adequate	11(10.0)	25 (23.1)	0.02
Adequate	110 (90.0)	83 (76.9)	
Non-Orthopedic Surgical			
Not Adequate	32 (29.1)	23 (22.1)	0.31
Adequate	78 (70.9)	81 (77.9)	

## Data Availability

Data is unavailable due to privacy and ethical restrictions.

## Abbreviations

The following abbreviations are used in this manuscript:

VTE	Venous Thromboembolism
DVT	Deep Vein Thrombosis
PE	Pulmonary Embolism
BMI	Body Mass Index
INR	International Normalized Ratio
SPSS	Statistical Package for the Social Sciences
CAAE	Brazilian Ethical Review Registration System
